# BertADP: a fine-tuned protein language model for anti-diabetic peptide prediction

**DOI:** 10.1186/s12915-025-02312-w

**Published:** 2025-07-15

**Authors:** Xueqin Xie, Changchun Wu, Yixuan Qi, Shanghua Liu, Jian Huang, Hao Lyu, Fuying Dao, Hao Lin

**Affiliations:** 1 The Clinical Hospital of Chengdu Brain Science Institute, School of Life Science and Technology, University of Electronic Science and Technology of China, Chengdu, 610054 China; 2 School of Biological Sciences, Nanyang Technological University, Singapore, 639798 Singapore

**Keywords:** Anti-diabetic peptides, Protein language models, Fine-tuning, Bioactive peptide prediction, Deep learning

## Abstract

**Background:**

Diabetes is a global metabolic disease that urgently calls for the development of new and effective therapeutic agents. Anti-diabetic peptides (ADPs) have emerged as a research hotspot due to their therapeutic potential and natural safety, representing a promising class of functional peptides for diabetic management. However, conventional computational approaches for ADPs prediction mainly rely on manually extracted sequence features. These methods often lack generalizability and perform poorly on short peptides, thereby hindering effective ADPs discovery.

**Results:**

In this study, we introduce a fine-tuning strategy of large-scale pre-trained protein language models (PLMs) for ADPs prediction, enabling automated extraction of discriminative semantic representations. We established the most comprehensive ADPs dataset to date, comprising 899 rigorously curated non-redundant ADPs and 67 newly collected potential candidates. Based on three model construction strategies, we developed 11 candidate models. Among them, BertADP (a fine-tuned ProtBert model) demonstrated superior performance in the independent test set, outperforming existing ADPs prediction tools with an overall accuracy of 0.955, sensitivity of 1.000, and specificity of 0.910. Notably, BertADP exhibited remarkable sequence length adaptability, maintaining stable performance across both standard and short peptide sequences.

**Conclusions:**

BertADP represents the first PLMs-based intelligent prediction tool for ADPs, whose exceptional identification capability will significantly accelerate anti-diabetic drug development and facilitate personalized therapeutic strategies, thereby enhancing precision diabetes management. Furthermore, the proposed approach provides a generalizable framework that can be extended to other bioactive peptide discovery studies, offering an innovative solution for bioactive peptide mining.

**Supplementary information:**

The online version contains supplementary material available at 10.1186/s12915-025-02312-w.

## Background

Diabetes has emerged as one of the fastest-growing chronic metabolic disorders worldwide. According to the International Diabetes Federation, the global diabetic population reached 537 million in 2021 and is projected to exceed 780 million by 2045 [1]. While current therapies including insulin and oral hypoglycemic agents (e.g., metformin) are widely used, their long-term application leads to adverse effects such as hypoglycemia and gastrointestinal disturbances [2–5]. These limitations underscore the urgent need for novel therapeutic strategies. Bioactive peptides have emerged as a new paradigm in drug development due to their unique advantages of high target specificity and low cytotoxicity [6]. Remarkable clinical advancements have been made with anti-diabetic peptides (ADPs), particularly glucagon-like peptide-1 (GLP-1) analogs. Studies have demonstrated that liraglutide reduces glycated hemoglobin (HbA1c) while significantly lowering cardiovascular risks [7], whereas semaglutide significantly improved beta cell function [8]. However, conventional experimental approaches for screening ADPs are inherently time-consuming and costly, hindering the efficient discovery and evaluation of novel candidates [9, 10].

The convergence of artificial intelligence and biology is revolutionizing bioactive peptide screening through data-driven approaches [11–14]. Computational high-throughput prediction methods, which decode sequence-function relationships of known bioactive peptides, enable rapid identification of potential candidates from millions of molecules, reducing experimental timelines and costs [15–17]. Machine learning (ML) methods have shown great promise in identifying ADPs, offering new strategies for the rational design and targeted development of functional peptides. For instance, Chen et al. developed AntiDMPpred [18], a classifier based on the random forest (RF) algorithm for ADP identification. However, the negative samples used in this model were collected from AVPdb, which had low probability of non-ADPs. Furthermore, the absence of independent external testing datasets raises concerns about the generalizability. Basith et al. proposed ADP-Fuse, which demonstrated strong performance in ADPs identification but was unable to predict short peptide sequences, thus narrowing its applicability [19]. Yue et al. applied deep learning techniques to offer new insights into ADPs prediction [20], yet their architecture lacked integration with advanced protein language models (PLMs).

Recent breakthroughs in PLMs are reshaping protein-related prediction task [21]. Large-scale self-supervised pre-trained models, such as evolutionary scale modeling (ESM) and ProtBert, have demonstrated outstanding performance across a variety of protein-related tasks, showcasing powerful sequence representation learning capabilities [22, 23]. By training on vast protein sequence datasets, these models are able to capture rich structural and functional information and exhibit strong generalization ability. Compared to conventional machine learning approaches, PLMs can automatically learn functionally relevant semantic representations and extract informative features, referred to as embeddings, without the need for handcrafted descriptors. Notably, embedding-based predictions are particularly advantageous when experimental data are limited [24], offering a novel and effective methodology for the prediction and classification for functional bioactive peptides.

**Fig. 1:**
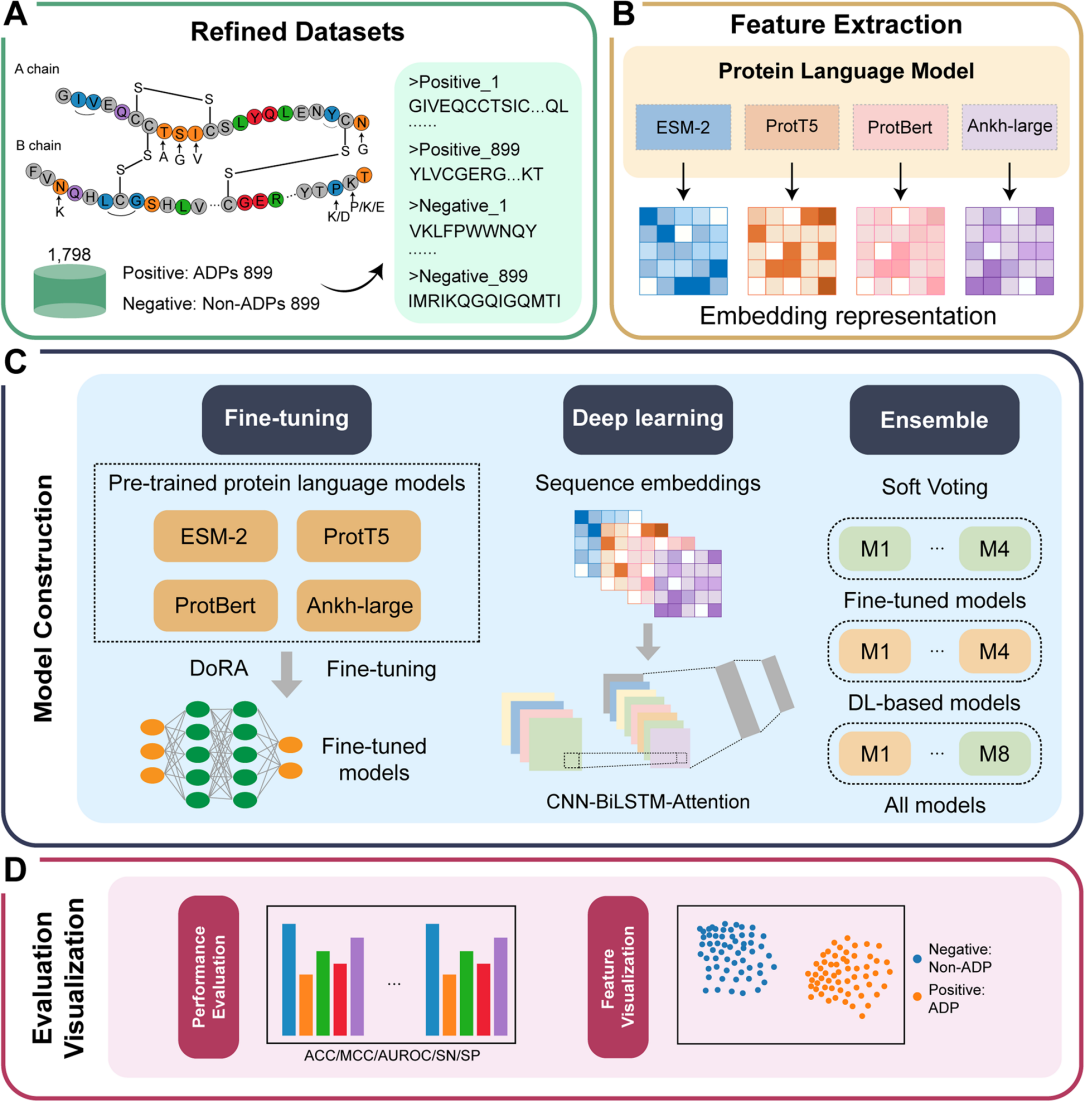
The overall schematic framework of BertADP. It includes data collection, embedding generation, model construction, and model evaluation. **A** Refined dataset construction. ADP sequences were collected from previously published studies. After merging these datasets and removing duplicates, 899 unique ADPs were retained as positive samples. An equal number of non-ADPs (899) were collected as negative controls. **B** Semantic feature extraction. Four pre-trained protein language models (PLMs) were used to extract sequence-specific semantic embeddings, generating four distinct types of representations. **C** Model construction. A total of 11 candidate models were trained using three different strategies: four fine-tuned PLMs models, four deep learning-based models trained separately using each of the four embeddings, and three ensemble models based on different integration strategies. **D** Model evaluation and feature visualization. The models were evaluated using eight performance metrics on independent test set. Additionally, t-distributed stochastic neighbor embedding (t-SNE) was applied to reduce the dimensionality of the embedding representations for visualization

In this study, we developed BertADP (fine-tuned ProtBert), the first fine-tuned pre-trained PLM-based tool for ADPs prediction (Fig. 1). We constructed a balanced benchmark dataset by integrating multi-source ADPs sequences, including newly curated potential ADPs from recent experimental studies. Four state-of-the-art PLMs (ESM2 [25], ProtT5 [26], Ankh [27], and ProtBert [26]) were systematically fine-tuned and evaluated for ADPs prediction tasks. The sequence embeddings generated by these PLMs were also fed into a hybrid deep learning architecture combining convolutional neural networks (CNN), bidirectional long short-term memory (BiLSTM), and an additive attention mechanism (CNN-BiLSTM-Attention). We further investigated ensemble strategies combining fine-tuned models with deep learning outputs. Experimental results demonstrated that the BertADP achieved optimal performance (ACC = 0.955) on the independent test set, outperforming deep learning-embedding models and the ensemble approach. Compared with existing methods, BertADP significantly improves ADPs prediction performance by more effectively capturing sequence features associated with anti-diabetic activity, thereby enabling accurate identification of ADPs.

## Results

### Amino acid composition and positional conservation analysis highlights distinct features of ADPs

**Fig. 2:**
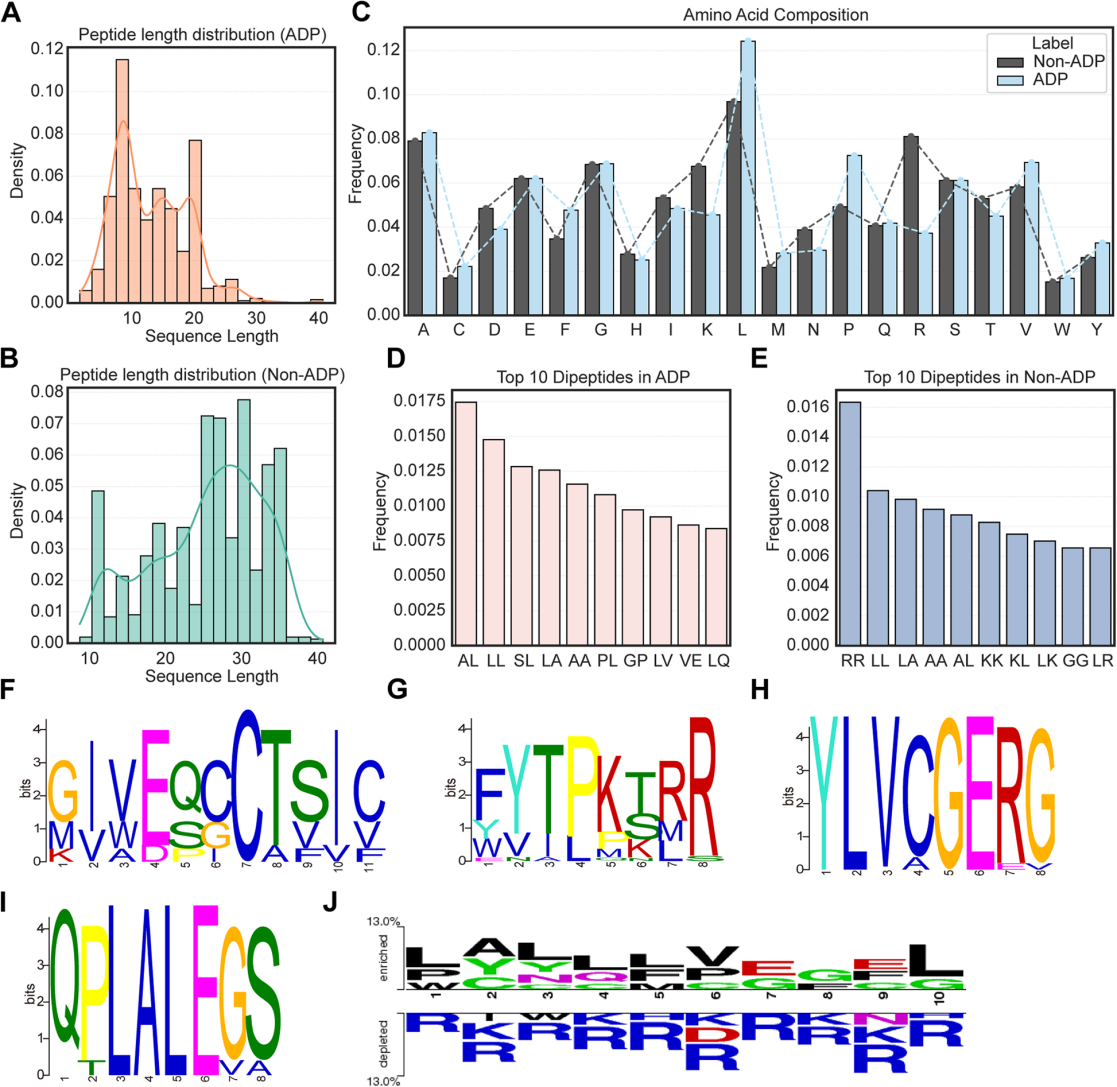
Compositional and positional conservation analysis. **A** Distribution of sequence lengths of ADPs in combined dataset. **B** Distribution of sequence lengths of non-ADPs in combined dataset. **C** A bar graph to represent percentage amino acid composition of ADPs and non-ADPs. **D** Top 10 dipeptides in ADPs. **E** Top 10 dipeptides in non-ADPs. **F**–**I** Conserved motifs of ADPs identified with MEME. **J** Positional conservation of the first five residues at the N-terminus and the last five residues at the C-terminus of ADPs versus non-ADPs

To explore potential differences in amino acid composition and positional conservation between ADPs and non-ADPs, we conducted systematic analysis on the integrated dataset. Sequence length distribution revealed that ADPs predominantly ranged from 6 to 22 amino acids (aa), accounting for 90.17% of all ADPs (Fig. 2A). The longest ADP contained 41 aa while the shortest comprised merely 2 aa. The dataset included 41 short peptides (< 6 aa), consisting of 20 pentapeptides and 21 shorter sequences, which enabled an evaluation of the model’s generalization ability across peptides of different lengths. Non-ADPs samples exhibited lengths concentrated between 18 and 35 aa, approximating a normal distribution (Fig. 2B).

The global frequency distribution of 20 standard amino acids revealed significant enrichment of hydrophobic/non-polar residues, such as Leu, Phe, Pro, Val, Met, and Ala, in ADPs, with Leu and Pro showing particularly prominent abundance (Fig. 2C). This hydrophobicity may facilitate ADPs penetration through lipid bilayers (e.g., cell membranes or lipoprotein surfaces) or enhance receptor binding via interactions with hydrophobic targets (e.g., transmembrane domains of insulin receptors or hydrophobic pockets of GLP-1 receptors). The high Pro content in ADPs is consistent with previous experimental findings that Pro-rich, well-designed peptides exhibit strong anti-diabetic activity [28]. Characteristic residues Pro and Cys in ADPs may confer structural rigidity through disulfide bond formation, potentially prolonging peptide half-life for sustained therapeutic effects. Non-ADPs predominantly contained charged residues (e.g., Asp, Lys, Arg, His) and hydrophilic/polar residues such as Asp, His, Lys, Asn, and Arg (Fig. 2C). Global statistics demonstrated that ADPs are dominated by charge-neutral/hydrophobic/non-polar residues, whereas non-ADPs favor charged/hydrophilic/polar residues. Dipeptide composition analysis identified AL, LL, SL, LA, and AA as the most frequent aa pairs in ADPs (Fig. 2D), with many of these containing Leu residues, underscoring their critical role. In contrast, non-ADPs predominantly featured RR, LL, LA, AA, AL, and KK (Fig. 2E).

Subsequently, we performed sequence conservation analysis on ADPs using the MEME tool [29] and identified four distinct motifs (Fig. 2F–I). The GIVEQCCTSIC motif precisely matches the N-terminal sequence of human insulin A-chain, forming a core conserved region critical for structural stability and functional activity of insulin analogs. The FYTPKT fragment within the FYTPKTRR motif resides in the C-terminal tail of insulin B-chain, while the YLVCGERG motif is embedded within the B-chain. Notably, the QPLALEGS motif shares homology with C-peptide fragments, which serve not only as pancreatic β-cell function markers but also play significant roles in diabetic management [30]. To further investigate sequence preferences in functional regions, we employed the Two Sample Logo tool [31] to compare positional amino acid conservation at N/C-termini between ADPs and non-ADPs (*t*-test, *p* value < 0.05) (Fig. 2J). Results demonstrated Leu residues dominated both termini of ADPs. Other amino acids showing high occurrence in ADPs at the N-termini included Pro and Trp at position 1; Ala, Tyr, and Cys at position 2; Tyr and Asn at position 3; Gln and Gly at position 4; and Phe and Met at position 5. In the C-terminal region of ADPs, Val, Pro, and Gly are enriched at position 6; Glu and Gly at positions 7 and 9; and Leu and Gly at position 10. Non-ADPs are more enriched in charged residues such as Arg at both termini. This positional conservation pattern may reflect ADPs functional requirements: hydrophobic residues (e.g., Leu, Phe, and Val) forming stable cores at termini, complemented by polar/charged residues (e.g., Gln, Glu, Tyr) mediating target interactions. In summary, these specific sequence preferences provide important insights for the rational design and functional prediction of anti-diabetic peptides.

### Evaluation of fine-tuning protein language models for ADPs prediction

**Fig. 3:**
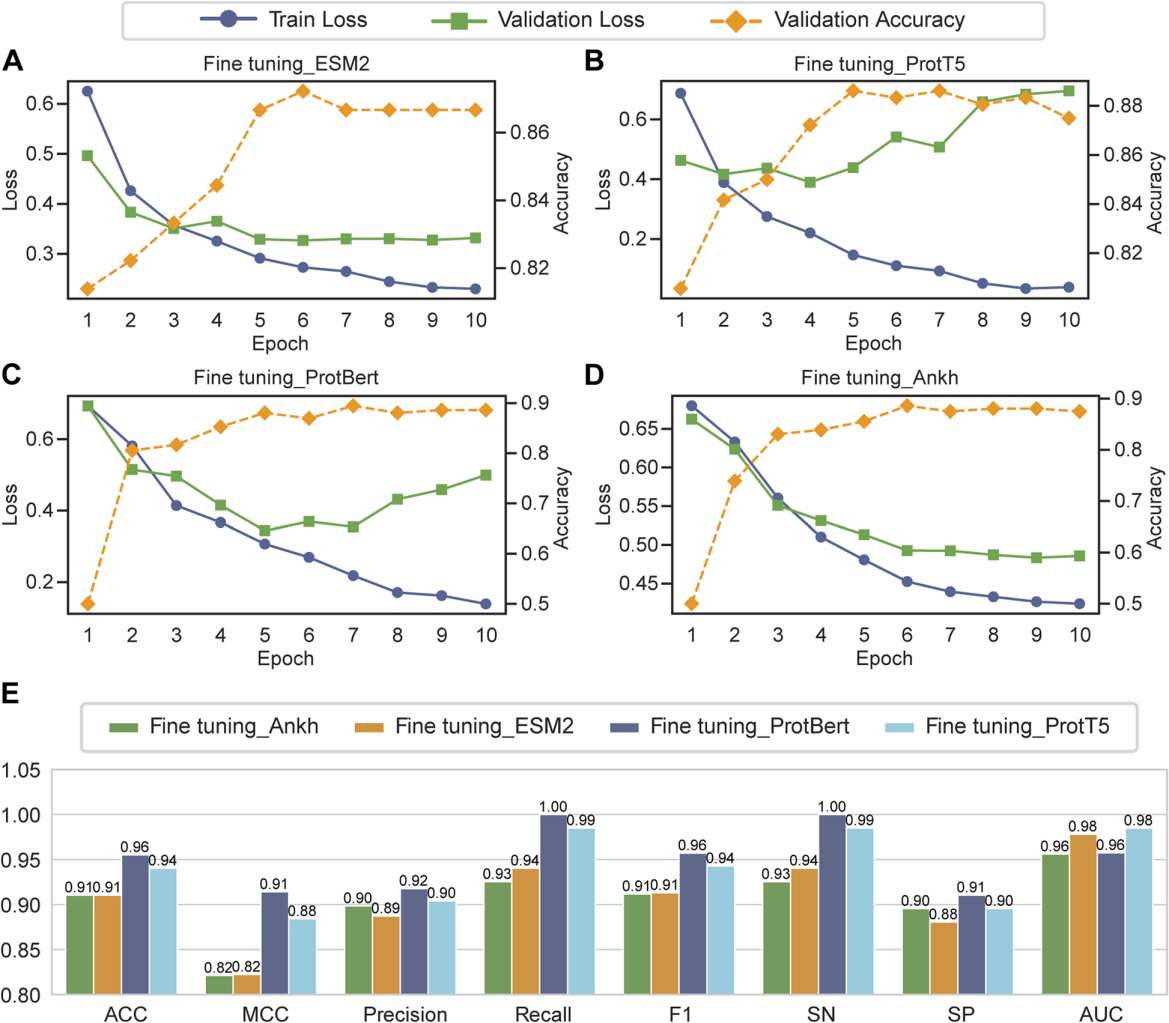
Performance comparison of fine-tuned PLMs. **A**–**D** Training loss curves, validation loss curves, and validation accuracy curves during the fine-tuning process of different PLMs. **E** Comprehensive performance comparison of fine-tuned PLMs on the independent test set across eight evaluation metrics

To evaluate the fine-tuning performance and generalization capacity of various PLMs for ADPs prediction, we examined the training and validation loss trajectories of four PLMs (Fig. 3A–D). All models exhibited continuously decreasing training loss with progressive convergence within 10 training epochs, indicating satisfactory model convergence. For validation loss, the ESM2 and Ankh-based models showed a synchronous decline with the training loss and gradually stabilized (Fig. 3A, D). While validation loss generally decreased during the early training phase, slight increases were observed in the ProtT5 and ProtBert fine-tuned models after several epochs. Specifically, the validation loss of the ProtT5 fine-tuned model began to rise from epoch 3, followed by fluctuating increases and decreases (Fig. 3B), whereas the ProtBert fine-tuned model’s validation loss started to increase from epoch 6 (Fig. 3C). Nevertheless, despite these fluctuations, the overall validation accuracy of these models remained relatively stable or improved throughout training, demonstrating the robustness of the fine-tuned models. The model checkpoint corresponding to the highest validation accuracy was selected as the final model for downstream evaluation. Compared to ESM2 and ProtT5, the Ankh and ProtBert models achieved higher accuracy on the validation set, with ProtBert additionally showing lower loss values, reflecting better learning performance.

In the independent test set (Fig. 3E), the BertADP (fine-tuned ProtBert) achieved optimal performance in seven out of eight evaluation metrics: ACC of 0.96, MCC of 0.91, precision of 0.92, recall/SN of 1.00, F1 score of 0.96, and SP of 0.91. These results indicated that the BertADP serves as a highly accurate and robust tool for predicting anti-diabetic peptides.

To further validate the superior performance of BertADP, we extracted four types of pre-trained semantic embeddings and used them to build predictive models with traditional machine learning algorithms (Additional file 1: Fig. S1). The results clearly demonstrate that the BertADP model offers outstanding predictive performance.

### Multi-embedding deep learning framework for ADPs prediction

**Fig. 4:**
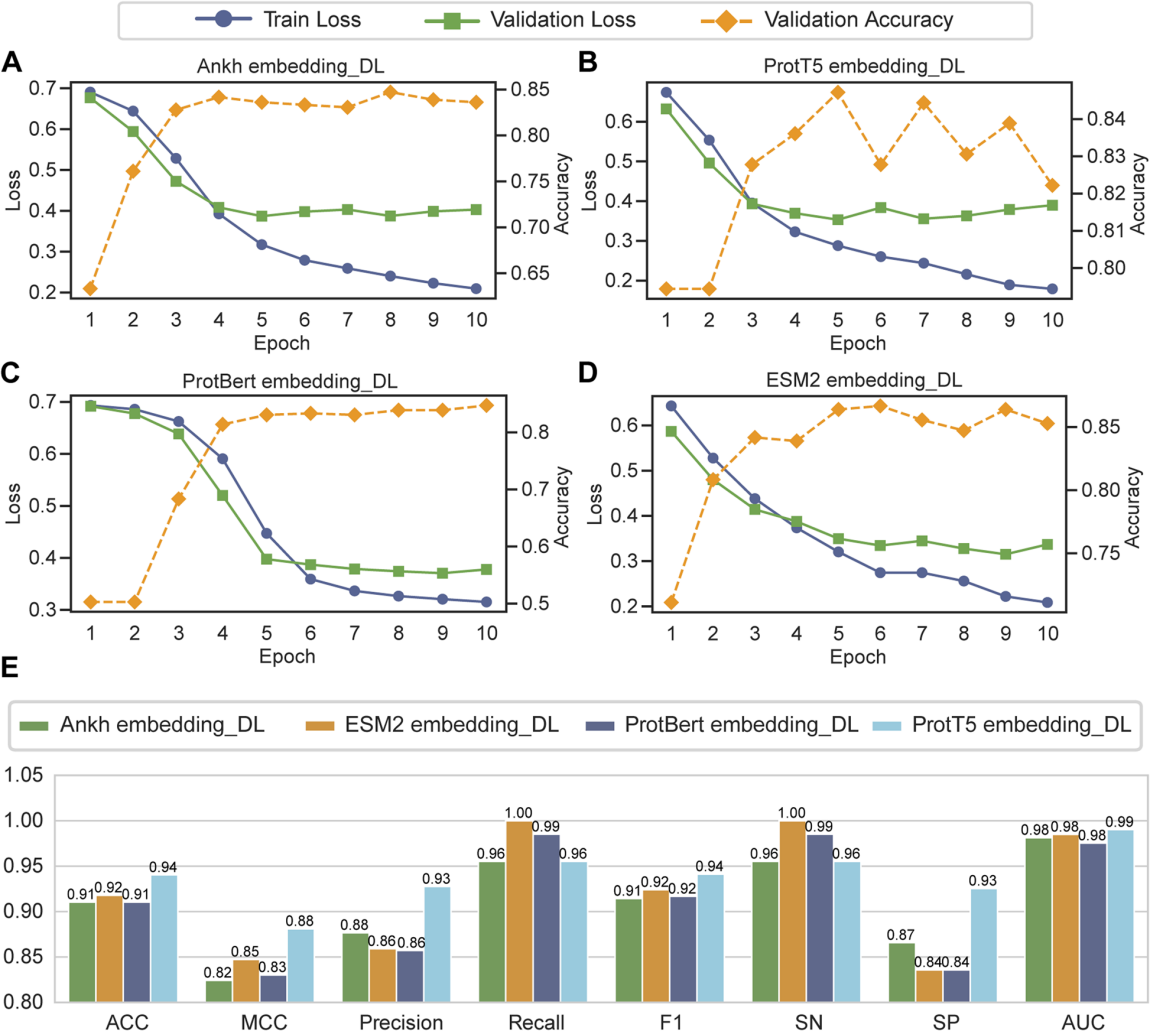
Performance comparison of deep learning models with diverse embeddings. **A**–**D** Training loss curves, validation loss curves, and validation accuracy curves during the training process of different deep learning models. **E** Comprehensive performance comparison of different embedding-based deep learning models on the independent test set across eight evaluation metrics

To investigate whether complex architectures could enhance ADPs prediction capabilities, we extracted multi-dimensional embeddings from four PLMs. These embeddings were fed into a unified CNN-BiLSTM-Attention framework, yielding four deep learning-based models. The training loss curves shown in Fig. 4A–D revealed that both training and validation losses for models decreased steadily and converged within 10 epochs. The optimal models were selected based on validation accuracy as previously described. Among them, the models based on ESM2 and ProtT5 embeddings demonstrated lower losses and higher validation accuracies.

In the independent test set evaluation, all embedding-based models achieved competitive performance across eight metrics: ACC, MCC, precision, recall, F1, SN, SP, and AUC. Notably, the ProtT5-embedded model exhibited superior performance in most metrics. Its ACC, MCC, precision, F1 score, SP, and AUC reached 0.94, 0.88, 0.93, 0.94, 0.93, and 0.99 (Fig. 4E), respectively, indicating strong generalization ability and predictive performance.

### Hierarchical ensemble strategy and systematic model evaluation highlight BertADP for optimal ADPs function prediction

**Fig. 5:**
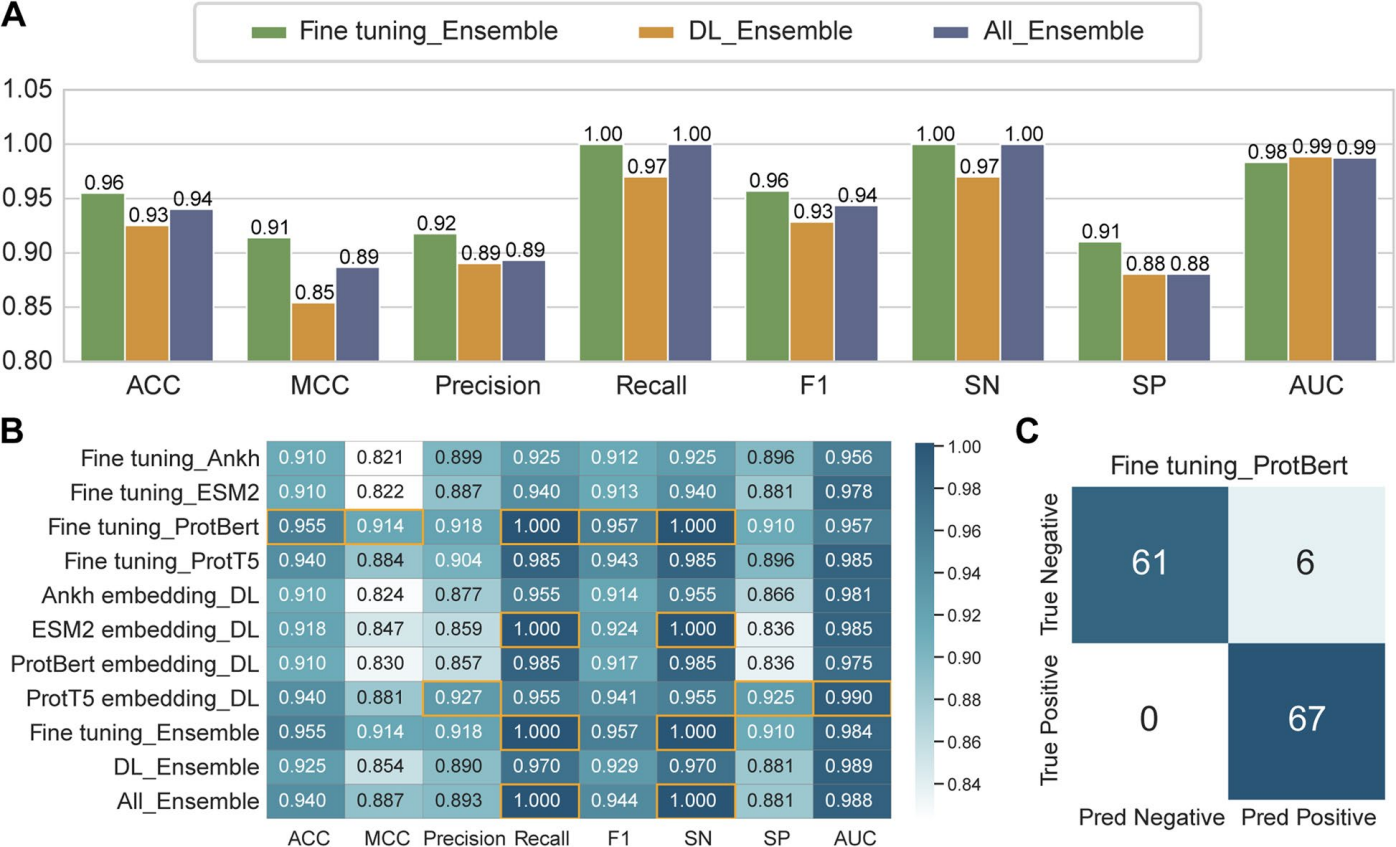
Ensemble strategies and cross-model selection of the optimal predictor. **A** Comprehensive performance comparison of three ensemble models on the independent test set across eight evaluation metrics. **B** Overall performance comparison of 11 candidate models, including 4 fine-tuned models, 4 embedding-based models, and 3 ensemble models, on the independent test set across eight evaluation metrics. Orange boxes indicate the best-performing value under each metric. **C** Confusion matrix of predictions on 134 independent test samples generated by the BertADP (fine-tuned ProtBert)

To systematically explore the synergistic potential among models, we designed a hierarchical ensemble framework. First, the four fine-tuned PLMs (ProtBert, ESM2, Ankh, ProtT5) were integrated to form the Fine-tuning Ensemble model. Second, the four deep learning (DL) models (CNN-BiLSTM-Attention framework) based on four PLMs embeddings were combined to create the DL Ensemble model. Finally, both types of ensemble models were further merged to construct the unified All Ensemble model. The three ensemble models showed comparable performance across various metrics. Among them, the Fine-tuning Ensemble slightly outperformed the others in ACC (0.96), MCC (0.91), precision (0.92), F1 score (0.96), SN/recall (1.00), and SP (0.91), although its AUC (0.98) was slightly lower (Fig. 5A).

We conducted a comprehensive comparison of 11 candidate models (4 fine-tuned models, 4 embedding-based models, and 3 ensemble models) across eight evaluation metrics to identify the optimal model (Fig. 5B). The BertADP (fine-tuned ProtBert) exhibited the most balanced predictive performance, achieving the best results in four key metrics: ACC (0.955), MCC (0.914), SN/recall (1.000), and F1 score (0.957) (Fig. 5B, C). Although the deep learning model based on ProtT5 embeddings achieved the highest AUC (0.990), considering the need for well-rounded performance in prediction tasks, the BertADP was ultimately selected as the optimal solution. Notably, although ensemble models integrate multiple types of models, they did not demonstrate significant performance improvements over individual models. For instance, the Fine-tuning Ensemble model achieved the same performance as BertADP in seven out of eight metrics, with only a slightly higher AUC (0.984). However, ensemble strategies have more computational complexity and require longer training times. Therefore, this study recommends BertADP as the preferred solution for lightweight, real-time prediction tasks, while hybrid ensembles are reserved for drug discovery scenarios with ultra-low tolerance for missed detections.

### BertADP outperforms existing ADPs prediction tools in rigorous benchmarking

**Fig. 6:**
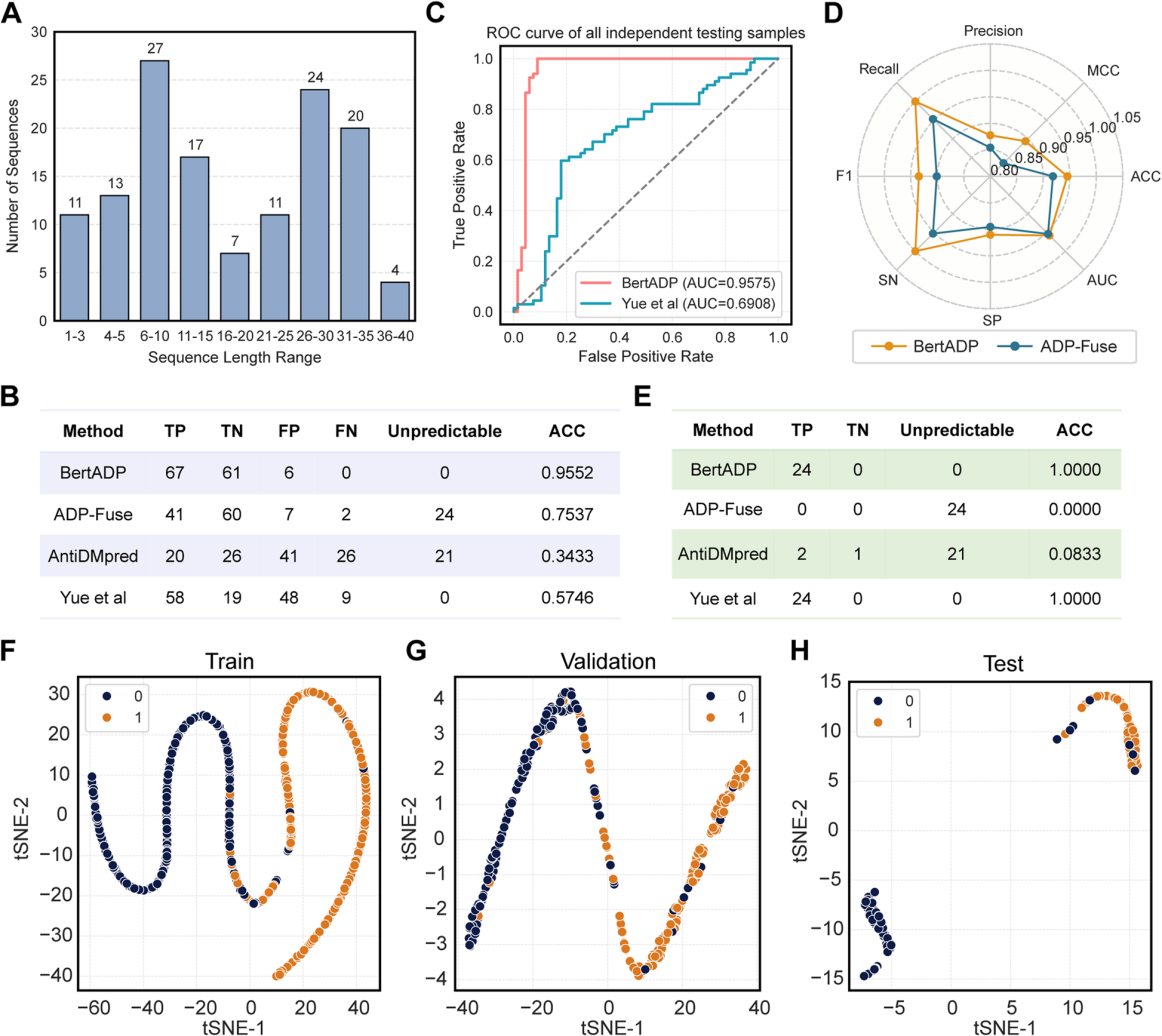
Comparison between BertADP and previously published tools. **A** Length distribution of 134 sequences in the independent test set. **B** Performance comparison of BertADP with other prediction tools on the entire test set (134 peptides). **C** ROC curve comparison between BertADP and the deep learning model proposed by Yue et al. on the complete test set. **D** Radar chart comparing BertADP and ADP-Fuse across eight evaluation metrics on the 110 samples with sequence lengths ≥ 6 amino acids. **E** Performance comparison between BertADP and other prediction tools on the 24 ADPs samples with sequence lengths < 6 amino acids. t-SNE visualizations of the 1024-dimensional embeddings generated by the ProtBert PLM for the training set (**F**), validation set (**G**), and independent test set (**H**). Labels “0” and “1” represent non-ADPs and ADPs, respectively

To evaluate the robustness and accuracy of BertADP compared to existing ADPs prediction tools, we conducted a comprehensive benchmarking using the independent test set. The independent test set consisted of 134 samples, including 67 ADPs and 67 non-ADPs. Most peptide sequences (27 samples) ranged from 6 to 10 aa in length, while 24 samples (all are ADPs) had sequences shorter than 6 aa, including 21 ADPs with sequences shorter than 5 aa (Fig. 6A). We compared BertADP with mainstream ADPs prediction methods, including ADP-Fuse [19], AntiDMPpred [18], and a deep learning model proposed by Yue et al. [20] (Additional file 1: Table S1). Among the 134 independent test peptides, ADP-Fuse failed to predict 24 short peptides (length < 6 aa), and AntiDMPpred could not process 21 sequences shorter than 5 aa (Fig. 6A, B). In contrast, both the methods by Yue et al. and BertADP successfully predicted all 134 samples without sequence length limitations. BertADP demonstrated superior ADPs identification performance across all 134 samples, achieving 100% accuracy for the 67 ADPs, with an overall ACC of 0.9552, AUC of 0.9575, and SN (recall) of 1.0000, significantly outperforming other models (Fig. 6B, C).

To ensure a fair comparison of different methods on consistent data, we divided the 134 samples into two subsets. The first subset consisted of 110 samples (sequence length ≥ 6 aa), which all methods could process, including 43 ADPs and 67 non-ADPs. The second subset consisted of the remaining 24 short ADPs (sequence length < 6 aa). Among the 110 comparable samples, BertADP achieved the optimal performance across all 8 evaluation metrics: AUC (0.9580), ACC (0.9455), SN/recall (1.0000), SP (0.9104), MCC (0.8938), precision (0.8776), and F1 score (0.9348) (Table 1, Fig. 6D), confirming its robustness and superiority in ADPs prediction tasks. Notably, ADP-Fuse also performed well in this subset, with an accuracy of 0.9182 (Table 1, Fig. 6D), ranking second.

**Table 1:** Comparative performance of ADPs prediction tools on the 110-sample subset

Method	AUC	ACC	SN/recall	SP	MCC	Precision	F1 score
BertADP	0.9580	0.9455	1.0000	0.9104	0.8938	0.8776	0.9348
ADP-Fuse	0.9540	0.9182	0.9535	0.8955	0.8353	0.8542	0.9011
AntiDMPpred	0.3783	0.4000	0.4186	0.3881	− 0.1892	0.3051	0.3529
Yue et al	0.6060	0.4818	0.7907	0.2836	0.0832	0.4146	0.5440

For the 24 short peptide ADPs, both BertADP and the method by Yue et al. achieved 100% prediction accuracy, highlighting their exceptional capability in short-sequence recognition (Fig. 6E). In summary, BertADP demonstrated excellent prediction capabilities for both standard and short sequences, with stable generalization performance and significant advantages over existing methods. These findings highlight its strong practical value and clinical translation potential, providing a reliable computational tool for the rational design of ADPs.

### The embedding space analysis confirms ProtBert’s discriminative power for ADPs

To investigate ProtBert’s capability in embedding representations for ADPs and non-ADPs sequences, we performed dimensionality reduction visualization using the t-SNE algorithm on 1024-dimensional sequence embeddings from the training, validation, and independent test sets (Fig. 6F–H). The results demonstrate that in the 2D reduced space, ADPs (positive samples) and non-ADPs (negative samples) clusters exhibit distinct distribution patterns with discernible separation boundaries between the clusters. This observation indicates that ProtBert effectively captures discriminative features related to anti-diabetic functionality during sequence encoding, thereby providing a robust and informative representation for downstream classification tasks. These findings comprehensively validate ProtBert’s effectiveness and applicability as a biological sequence feature extractor.

## Discussion

Diabetes, as a major global metabolic disorder, necessitates urgent development of therapeutic agents. ADPs, a class of bioactive molecules with potential therapeutic value, have demonstrated significant clinical prospects in diabetes treatment and management [32]. Accurate identification of ADPs not only facilitates peptide drug development but also provides theoretical support for mining functional peptides from food sources and natural proteins. Therefore, establishing efficient and accurate ADPs identification models holds crucial value for functional peptide design, peptide library screening, and related mechanistic studies.

Although several computational tools have been proposed for ADPs identification, most rely on manually extracted sequence features, resulting in limited generalizability and poor performance on special cases such as short peptides [18, 19]. In this study, we introduced the fine-tuning of large-scale pre-trained PLMs into ADPs prediction for the first time. We constructed the largest known ADPs dataset to date, integrating 899 non-redundant sequences from previous studies and additional 67 newly collected potential ADPs from the literature. Based on three modeling strategies, we built 11 candidate models, including four fine-tuned PLMs, four deep learning models using PLMs-based embeddings, and three ensemble models based on different strategies. All model training and inference were performed on a server equipped with two NVIDIA GeForce RTX 4090 GPUs (24 GB VRAM each). The training time for each model did not exceed 10 min. Comparative results revealed that the three ensemble models did not show substantial performance improvements over the eight individual models. Among them, the Fine-tuning Ensemble model achieved identical results to BertADP in seven out of eight metrics, with only a slightly higher AUC of 0.984. However, ensemble approaches typically require more computational resources and longer training times, limiting their feasibility in practical applications. Therefore, taking both predictive performance and real-world usability into account, we recommend BertADP as the preferred model for lightweight and real-time prediction tasks. Ensemble models may still be valuable in drug discovery settings where extremely low false negative rates are crucial and computational cost is less of a concern. BertADP strikes a practical balance between accuracy and efficiency, making it a more promising candidate for broad deployment. Further feature visualization analysis showed that BertADP can extract discriminative semantic embeddings directly from raw sequences, laying a solid foundation for downstream tasks.

To prevent overfitting of BertADP, we employed multiple strategies during the training process. First, the dataset was strictly divided into a training set, a validation set, and an independent test set to ensure the reliability and generalizability of model evaluation. Second, we manually selected the optimal number of training epochs. Specifically, after each epoch, we recorded the training loss, validation loss, and validation accuracy to assess signs of overfitting and determine the most appropriate stopping point. The superior performance of BertADP on the independent test set indicates that the model not only performs well on training data but also generalizes effectively to unseen samples. In summary, these strategies collectively ensured that BertADP maintained robust and stable predictive performance.

In comparative studies, only the deep learning framework proposed by Yue et al. [20] and the BertADP successfully predicted all samples in the independent test set (including short peptides), without being affected by sequence length constraints. Both methods achieved 100% accuracy on 24 peptides shorter than 6 amino acids. Notably, Yue’s approach also utilized embeddings from a PLM (SEM2) combined with convolutional neural networks. This indicates that PLM-derived feature representations possess superior generalizability, effectively capturing structural and functional information of short peptides while overcoming traditional methods’ length dependency and performance limitations. Short peptides have attracted increasing attention in therapeutic drug development in recent years due to their advantages such as high synthetic efficiency, superior stability, and low immunogenicity [33]. Compared to traditional proteins or longer peptide drugs, short peptides are better suited for large-scale synthesis and rapid screening, thereby possessing greater translational potential. The robust predictive performance of BertADP on short peptides further underscores its applicability in the screening and design of therapeutic peptides.

This study still has certain limitations. Firstly, although the constructed dataset is more comprehensive than previous efforts, the overall sample size remains limited due to the scarcity of known anti-diabetic peptides. Secondly, the model’s efficacy and safety in real clinical settings require further experimental validation and clinical trials. Additionally, the current model primarily relies on sequence information; future work could integrate multi-dimensional biological data and disease mechanisms to develop more targeted predictive models. As more high-quality ADPs are discovered and accumulated, we plan to continuously expand the dataset to improve the model’s generalizability and robustness. Future research may also focus on designing therapeutic peptide prediction models specific to different types of diabetes, such as immunomodulatory peptides for type 1 diabetes (T1D) and insulin-sensitizing peptides for type 2 diabetes (T2D), thereby advancing precision medicine and enhancing clinical translational potential.

## Conclusions

In this study, we developed BertADP (fine-tuned ProtBert), the first fine-tuned pre-trained PLM-based ADPs prediction tool. BertADP demonstrated excellent performance on the independent test set, achieving 100% accuracy in identifying 67 ADPs and overcoming sequence length limitations in short peptide prediction (length < 6 aa), with significantly superior performance (ACC = 0.955, SN = 1.000, SP = 0.910) over existing models. This tool could accelerate anti-diabetic peptide discovery and reduce experimental costs. Given the strong generalization capacity of PLMs across diverse protein tasks, the modeling strategy adopted in BertADP also shows promising transferability to the prediction of other functional peptides.

## Methods

### Benchmark dataset construction

This study employed a multi-source integration strategy to build benchmark datasets. We integrated ADPs datasets curated by Chen et al. [18], Basith et al. [19], and Yue et al. [20], all of which collected ADPs associated with type 1 (T1D) and type 2 diabetes (T2D) from the BioDADPep database [34]. After merging these datasets and removing duplicates, a total of 899 unique ADPs were retained as positive samples. For negative samples, we adopted the dataset constructed by Basith et al., which includes random peptides and bioactive peptides with antimicrobial activities, such as anticancer and antihypertensive properties. To construct a balanced dataset with a 1:1 ratio of positive to negative samples, we randomly extracted additional negative samples from the independent test set to supplement the dataset. Then, the dataset was further split into 80% for training and 20% for validation.

Additionally, we collected 67 novel non-redundant peptides reported to possess potential anti-diabetic activity, carefully filtered to ensure no overlap with the training dataset. Several have been experimentally confirmed to ameliorate metabolic dysfunctions linked to diabetes [35, 36]. To ensure fairness and evaluate model robustness, another 67 negative samples were randomly selected from the remaining negative pool of Basith et al.’s independent test dataset to form a newly external independent test set.

### Embedding generation via protein language models

The PLMs, including ESM2 [25], ProtT5 [26], Ankh [27], and ProtBert [26], were employed to generate distinct embedding representations. As shown in Additional file 1: Table S2, these pre-trained models differed in size and architecture, ranging from 420 million parameters to 3 billion parameters. The ESM2 PLM is an encoder-based model built on the RoBERTa architecture and trained using an unsupervised masked language modeling objective [25]. Specifically, the version with 3 billion parameters (esm2_t36_3B_UR50D) was used as the pre-trained model in this study. ProtT5 and Ankh are both built on the T5 architecture [37], using the prot_t5_xl_uniref50 and ankh-large versions, respectively. ProtBert is based on the BERT model and was pre-trained in a self-supervised manner on large-scale protein sequence corpora. The prot_bert version were utilized for further analysis. All models were initialized with pre-trained checkpoints available from Huggingface. Based on these four PLMs, we obtained four distinct types of embedding representations.

### Model construction

This study employed three modeling strategies: fine-tuned PLMs, embedding-based deep learning models, and ensemble models.

### Fine-tuning protein language models

Pre-trained PLMs based on the Transformer architecture excel at learning rich semantic and structural information from large-scale unsupervised protein sequences, demonstrating superior generalization in various downstream biological tasks. We fine-tuned four representative PLMs (ESM2, ProtT5, Ankh, and ProtBert) for binary classification of ADPs. The fine-tuning process utilized the weight-decomposed low-rank adaptation (DoRA) method [38], a parameter-efficient strategy that freezes most of pre-trained parameters and introduces low-rank weight matrices only in critical submodules (e.g., query and key-value matrices in attention mechanisms). This approach significantly reduces computational costs while enhancing generalization under limited data and prevents catastrophic forgetting [39]. Training followed a standard supervised paradigm: the training set updated parameters, while the validation set was used to monitor performance and select the best model weights. The training was conducted over 10 epochs, with training loss, validation loss, and validation accuracy recorded each epoch. The best-performing epoch based on validation accuracy was selected as the optimal model.

### Embedding-based deep learning framework

After obtaining high-dimensional embeddings from the four PLMs, we designed a multi-stage deep learning architecture integrating 1D CNN, BiLSTM, and additive attention mechanisms (CNN-BiLSTM-Attention) [40]. The 1D CNN module extracts local feature patterns from the input embeddings by applying convolutional filters of varying kernel sizes and numbers. These features are then passed to a BiLSTM module, which captures global contextual dependencies. The output of the BiLSTM was further weighted using an additive attention mechanism, yielding a global representation vector that emphasizes the critical features for anti-diabetic activity prediction. A final fully connected layer performs the binary classification to distinguish between ADPs and non-ADPs. The model was trained using the cross-entropy loss function and the Adam optimizer. Training was conducted for 10 epochs, with the best model selected based on validation accuracy.

### Ensemble strategy

We applied a soft voting strategy to combine the predictions of different models, averaging the predicted probabilities for each sample across models, and assigning the final label based on the highest average probability. Three ensemble models were constructed: (1) an ensemble of the four fine-tuned models, (2) an ensemble of the four CNN-BiLSTM-Attention models, and (3) a hybrid ensemble of all eight models. This approach integrates the strengths of various models in feature extraction and classification strategies.

### Model evaluation

During the training of both the fine-tuned models and the deep learning framework-based models, the cross-entropy loss function was used to compute the loss on the training and validation sets. In addition, several evaluation metrics were employed to comprehensively assess model performance [41, 42], including accuracy (ACC), sensitivity (SN/recall), specificity (SP), precision, F1 score, and Matthews Correlation Coefficient (MCC).

1 $$\begin{array}{c}\left\{\begin{array}{c}ACC=\frac{\text{TP}+\text{TN}}{\text{TP}+\text{TN}+\text{FP}+\text{FN}}\\ SN \left(\text{recall}\right)=\frac{\text{TP}}{\text{TP}+\text{FN}}\\ SP=\frac{\text{TN}}{\text{TN}+\text{FP}}\\ precision=\frac{\text{TP}}{\text{TP}+\text{FP}}\\ F1 score=2\times \frac{\text{precision}\times \text{recall}}{\text{precision}+\text{recall}}\\ MCC=\frac{\text{TP}\times \text{TN}-\text{FP}\times \text{FN}}{\sqrt{\left(\text{TP}+\text{FN}\right)\times \left(\text{TN}+\text{FN}\right)\times \left(\text{TP}+\text{FP}\right)\times \left(\text{TN}+\text{FP}\right)}}\end{array}\right.\end{array}$$

where TP, FP, TN, and FN denote the number of true positives, false positives, true negatives, and false negatives, respectively.

Additionally, the receiver operating characteristic (ROC) curve and the area under the curve (AUC) were also used to quantitatively assess the model’s ability to distinguish ADPs from non-ADPs.

## Supplementary information

Additional file 1: BertADP: A fine-tuned protein language model for anti-diabetic peptide prediction, Fig. S1 and Tables S1 and S2. Fig. S1 Overall performance comparison between BertADP and traditional machine learning-based models with different embedding representations on the independent test set across eight evaluation metrics. Table S1 Comparison between BertADP and existing ADPs prediction tools. Table S2 Details of protein language models applied in the study.

## Data Availability

All code and data generated or analyzed during this study are included in this published article, its supplementary information files, and publicly available repositories: https://github.com/xiexq007/BertADP and 10.5281/zenodo.15675400 [43].

## Abbreviations

ADPs: Anti-diabetic peptides
PLMs: Protein language models
GLP-1: Glucagon-like peptide-1
HbA1c: Glycated hemoglobin
ML: Machine learning
DL: Deep learning
RF: Random forest
ESM: Evolutionary scale modeling
CNN: Convolutional neural networks
BiLSTM: Bidirectional long short-term memory
t-SNE: T-distributed stochastic neighbor embedding
aa: Amino acids
T1D: Type 1 diabetes
T2D: Type 2 diabetes
DoRA: Weight-decomposed low-rank adaptation
ACC: Accuracy
SN: Sensitivity
SP: Specificity
MCC: Matthews correlation coefficient
ROC: Receiver operating characteristic
AUC: Area under the curve
