# Supplementary material for: BertADP: a fine-tuned protein language model for anti-diabetic peptide prediction
Source: BMC Biol. 2025 Jul 15;23:210. doi: 10.1186/s12915-025-02312-w (PMC12261731; doi:10.1186/s12915-025-02312-w)
Supplement: Supplementary file 1 — Additional file 1: BertADP: A fine-tuned protein language model for anti-diabetic peptide prediction, Fig. S1 and Tables S1 and S2. Fig. S1 Overall performance comparison between BertADP and traditional machine learning-based models with different embedding representations on the independent test set across eight evaluation metrics. Table S1 Comparison between BertADP and existing ADPs prediction tools. Table S2 Details of protein language models applied in the study. [file 12915_2025_2312_MOESM1_ESM.docx]

**Supplementary material for**

**BertADP: A fine-tuned protein language model for anti-diabetic peptide prediction**

Xueqin Xie^1,3^, Changchun Wu^1,3^, Yixuan Qi^1^, Shanghua Liu^1^, Jian Huang^1^, Hao Lyu^1,*^, Fuying Dao^2,*^, Hao Lin^1,*^

^1^ The Clinical Hospital of Chengdu Brain Science Institute, School of Life Science and Technology, University of Electronic Science and Technology of China, Chengdu, 610054, China.

^2^ School of Biological Sciences, Nanyang Technological University, Singapore, 639798, Singapore.

^3^ Authors contributed equally to this work.

^*^ Corresponding authors:

hao.lyu@uestc.edu.cn (Hao Lyu)

fuying.dao@ntu.sg.edu (Fuying Dao)

hlin@uestc.edu.cn (Hao Lin)

**Supplementary figures**


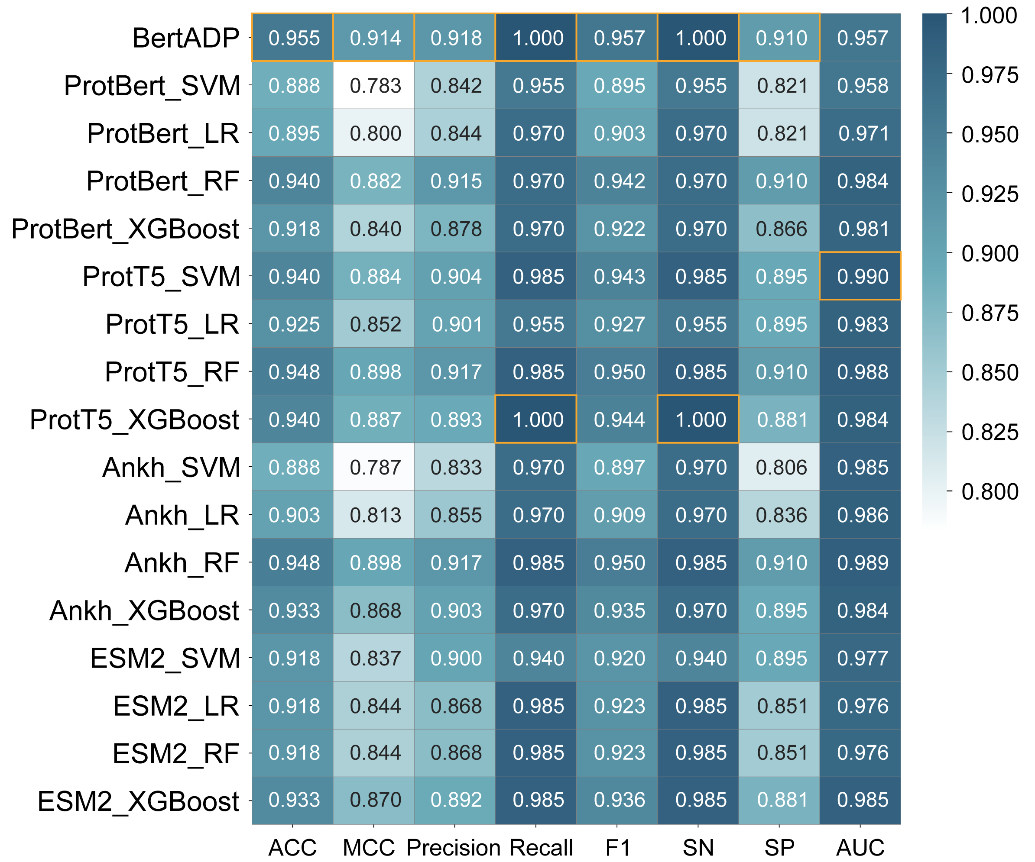


**Fig. S1** Overall performance comparison between BertADP and traditional machine learning-based models with different embedding representations on the independent test set across eight evaluation metrics. Orange boxes indicate the best-performing value under each metric.

**Supplementary tables**

**Table S1** Comparison between BertADP and existing ADPs prediction tools

| Method | Feature extraction | Model architecture | Usability |
| --- | --- | --- | --- |
| ADP-Fuse | Handcrafted feature descriptors | Tree-based stacking ensemble | Web server, length ≥ 6 aa |
| AntiDMpred | Handcrafted feature descriptors | Random forest | Web server, length ≥ 5 aa |
| Yue et al | PLMs embeddings | Deep learning | GitHub, no length limit |
| BertADP | PLMs embeddings | Fine-tuned PLMs | GitHub, no length limit |

PLMs: Protein language models; aa: amino acids.

**Table S2** Details of protein language models applied in the study

| **Model** | **Number of parameters** | **Encoder layers** | **Embedding size** | **Huggingface model checkpoint** |
| --- | --- | --- | --- | --- |
| ESM2 | 3000 M | 36 | 2560 | esm2_t36_3B_UR50D |
| ProtT5 | 1200 M | 24 | 1024 | prot_t5_xl_uniref50 |
| Ankh | 1900 M | 48 | 1536 | ankh-large |
| ProtBert | 420 M | 24 | 1024 | prot_bert |
